# Trend of disparity between coastland and inland in medical expenditure burden for rural inpatients with malignant tumor in southeast of China from 2007 to 2016

**DOI:** 10.1186/s12885-020-06769-6

**Published:** 2020-04-07

**Authors:** Rong Fu, Zheng Lin, Fei He, Yixian Jiang, Zhenquan Zheng, Zhijian Hu

**Affiliations:** 1 Department of Epidemiology and Health Statistics, Fujian Provincial Key Laboratory of Environment factors and Cancer, School of Public Health, Fujian Medical University, 1 Xuefu North Road, Fuzhou, 350122 Fujian Province China; 2 Institute of health research, Fujian Medical University, 1 Xuefu north Road, Fuzhou, 350122 Fujian Province China; 3 Fujian Digital Institute for Tumor Big Data, Fuzhou, 350122 China

**Keywords:** Medical expenditure burden, Trend, Disparity, Malignant tumor, New Rural Cooperative Medical Scheme

## Abstract

**Background:**

New Rural Cooperative Medical Scheme (NRCMS) was developed to improve the health security for rural residents. This study aimed to assess the trend of disparity between coastland and inland in medical expenditure burden for rural inpatients with malignant tumor from 2007 to 2016 under the effect of NRCMS.

**Methods:**

The data from medical records of 1,306,895 patients with malignant tumor who had NRCMS in 2932 hospitals was collected. The relative differences [95% confidence intervals (CIs)] between coastland and inland in four medical expense indicators were calculated using generalized linear models to assess the trend of disparity over time.

**Results:**

In total, there were 769,484 (58.88%) coastland patients and 537,411 (41.12%) inland patients. Male and patients aged older than 44 years accounted for 56.87 and 80% of 1,306,895 patients, respectively. After adjusting for gender, age, tumor site and hospital level, coastland patients had higher hospitalization expenses which were all medical expenses incurred during the hospitalization, lower reimbursement ratio and ratio of out-of-pocket expenses to disposable income than inland patients in most years. The surgery expenses of coastland patients were lower than those of inland patients in 2016. The relative differences (95% CIs) between coastland and inland in medical expense indicators were moving closer to 1.0 from 2007 to 2010 among patients without surgery, implying that the disparity between two areas significantly narrowed. The range of change was similar between two areas from 2011 to 2016 whether among patients without or with surgery, implying that the disparity did not significantly change. The disparity between coastland and inland depended on the household income situation. For low-income patients, the differences between two areas in medical expense indicators were not statistically significance in most cases and the disparity between two areas did not significantly change over time.

**Conclusions:**

Under the effect of NRCMS, the medical expenditure burden of rural inpatients reduced but suffering from malignant tumor was still catastrophic. As a whole, the inland patients had heavier medical expenditure burden than coastland patients. Because of economic factors and medical assistance policies, the medical expenditure burden was similar between coastland and inland low-income patients.

## Background

Fujian province located in the southeast of China has six coastal cities and three inland cities. Compared to the inland, the industry and agriculture in coastland are more developed due to the convenient transportation, fertile land and other advantages. Because coastal cities face Taiwan across the Taiwan Strait, a lot of Taiwan businessmen are attracted to invest that further promote the economic development of the coastland. According to the Statistical Yearbook of Fujian province, coastland rural residents had higher per capita annual disposable income than inland rural residents and the disparity between coastland and inland became slightly wider over time [1].

The latest data showed that the age-standardized incidence rate and age-standardized mortality rate by world standard population of malignant tumor in Fujian province was 192.28/100,000 and 123.91/100,000, respectively [2], which were both greater than national average level (186.53/100,000 and 106.09 /100,000, respectively) [3]. The malignant tumor had become the leading cause of death among Fujian residents which caused large disease burden [4, 5]. Now that there was the disparity between coastland and inland in economic, was there the disparity between two areas in disease economic burden of malignant tumor?

The State Council of China promulgated New Rural Cooperative Medical Scheme (NRCMS) in 2003, in order to improve the health security, reduce the medical burden, and solve the problems of illness-caused poverty and poverty-caused illness for rural residents [6, 7]. The NRCMS accompanied with Urban Employee Basic Medical Insurance and Urban Resident Basic Medical Insurance constituted the Basic Medical Insurance System in China [8–11]. The Fujian government selected three pilot counties to carry out the NRCMS in 2004 and achieved good effect [12]. Therefore, the NRCMS was expanded to the whole province in 2007 [13]. The participation rate of NRCMS increased from 85.05% in 2007 to 99.9% in 2015 [14]. More than 20 million Fujian rural residents benefited from this policy. Under the effect of NRCMS, did the disease economic burden of both coastland and inland rural patients with malignant tumor reduce? And how did the trend of the disparity between coastland and inland change over time? In this study, the data of medical expenses from the medical records of inpatients with NRCMS in Fujian Province from 2007 to 2016 was used to describe the medical expenditure burden of coastland and inland rural patients with malignant tumor and analyze the trend of the disparity between coastland and inland in medical expenditure burden over time. The findings will provide evidence for improving the medical insurance system and the equity in health service for Chinese rural residents.

## Methods

### Data source and study population

The data was extracted from the medical records of inpatients with NRCMS in Fujian Province between 1 January 2007 and 31 December 2016. The database managers in each hospital de-identified medical records by removing names, addresses and telephone numbers of patients before abstracting the data. Every patient was identified by a unique medical record number. To be eligible for this study, patients had to be diagnosed as malignant tumor according to International Classification of Diseases, Tenth Revision (ICD-10).

Between 1 January 2007 and 31 December 2016, all inpatients with malignant tumor who had NRCMS (1,323,291 patients) were entered into the study population. Patient variables included gender, age, residential county, name of disease and ICD-10 code (tumor site), name of admitted hospital, hospital level, surgery or not, medical expense indicators and household income situation (low-income or not). Low-income patients were defined as patients whose household per capita income were below the local minimum living standard. We excluded patients with missing values and/or illogical values. This resulted in a total of 1,306,895 patients in 2932 hospitals which included township, county, municipal and provincial hospitals. The patients were classified into coastland and inland patients according to their residential county.

### Medical expense indicators

Four medical expense indicators were used to assess the medical expenditure burden of patients with malignant tumor, as following: (1) Hospitalization expenses: all medical expenses incurred during the hospitalization; (2) Surgery expenses: the expenses for the operation technique during the hospitalization; (3) Reimbursement ratio: calculated as the reimbursement amount divided by hospitalization expenses; (4) Ratio of out-of-pocket expenses to disposable income (OOP ratio): calculated as out-of-pocket expenses divided by disposable income. The out-of-pocket expenses were the differences between hospitalization expenses and reimbursement amount. The disposable income was per capita annual disposable income of rural residents at county level where the patient lived, which was extracted from Fujian Statistics Bureau, Fujian Statistical Yearbook from 2008 to 2017 (Available from: http://tjj.fujian.gov.cn/xxgk/ndsj/).

To make the comparison of expenses across time meaningful, the amount of medical expenses from 2007 to 2016 were transformed by Consumer Price Index (CPI) to the price level in 2007. The transformation formula is following: real price = nominal price × (CPI of base year/CPI of object year). The CPI from 2007 to 2016 was displayed in Supplementary Table 1, reference as CPI = 100 in 1978 [15]. Therefore, the real price of 100 yuan (1$ = 7.0 yuan) which was nominal price was transformed to 94.4 yuan [100 yuan × (493.6/522.7)] in 2008, and so on (Supplementary Table 1).

### Statistical analysis

Numbers (percentages) were calculated for categorical variables. Means and standard deviations were calculated for four medical expense indicators: hospitalization expenses, surgery expenses, reimbursement ratio and OOP ratio. The disparity between coastland and inland in medical expenditure burden was defined as the relative differences between two areas in medical expense indicators, inland as the reference group. The relative differences [95% confidence intervals (CIs)] were calculated using generalized linear models with logarithmic link function and gamma distribution for each medical expense indicator from 2007 to 2016. The disparity between coastland and inland in medical expenditure burden was considered ‘widening’ if the difference was moving away from 1.0 and ‘narrowing’ if the difference became closer to 1.0 over time, regardless of whether initial value was greater or less than 1.0. Over time, if the 95% CIs of differences did not overlap, the change of disparity between coastland and inland was considered statistically significant [16, 17]. Stratified analyses by surgery or not and household income situation were conducted to determine whether the changes of disparity differed across strata. The gender, age, tumor site and hospital level were adjusted in all the multivariate analyses. All analyses were conducted using SAS version 9.4. *P* values for differences between two groups were not reported because of the large sample size.

## Results

### Demographic characteristics

Demographic characteristics of the study population are shown in Table 1. In total, there were 769,484 (58.88%) coastland patients and 537,411 (41.12%) inland patients. The proportion of male patients were more than that of female patients (56.87% VS 43.13%); 80% of 1,306,895 patients aged older than 44 years; Most patients (70.58%) were admitted in the hospitals above county level; The proportion of low-income patients in inland was 1.8 times than that in coastland (5.24% VS 2.91%); About 13% of 1,306,895 patients had undergone surgery; The proportions of patients with stomach/duodenum, lung/bronchia and esophagus cancer were the top three, accounting for 13.60, 13.10 and 9.99%, respectively.

**Table 1:** Demographic characteristics of coastland and inland rural patients with malignant tumor, n (%)

Variable		Coastland (*n* = 769,484)	Inland (*n* = 537,411)	Total (*n* = 1,306,895)
Gender	Male	450,264 (58.52)	293,007 (54.52)	743,271 (56.87)
	Female	319,220 (41.48)	244,404 (45.48)	563,624 (43.13)
Age, years	< 18	12,778 (1.66)	11,819 (2.20)	24,597 (1.88)
	18–44	132,622 (17.24)	104,170 (19.38)	236,792 (18.12)
	45–59	320,602 (41.66)	218,036 (40.57)	538,638 (41.22)
	60–74	244,884 (31.82)	162,648 (30.27)	407,532 (31.18)
	≥75	58,598 (7.62)	40,738 (7.58)	99,336 (7.60)
Hospital level	Township	61,882 (8.04)	53,813 (10.01)	115,695 (8.85)
	County	113,005 (14.69)	155,860 (29.00)	268,865 (20.57)
	Municipal	352,358 (45.79)	199,272 (37.08)	551,630 (42.21)
	Provincial	242,239 (31.48)	128,466 (23.90)	370,705 (28.37)
Low income	No	747,128 (97.09)	509,262 (94.76)	1,256,390 (96.14)
	Yes	22,356 (2.91)	28,149 (5.24)	50,505 (3.86)
Surgery	No	665,851 (86.53)	466,875 (86.87)	1,132,726 (86.67)
	Yes	103,633 (13.47)	70,536 (13.13)	174,169 (13.33)
Tumor site	Stomach, duodenum	108,714 (14.13)	68,992 (12.84)	177,706 (13.60)
	Lung, bronchia	100,031 (13.00)	71,178 (13.25)	171,209 (13.10)
	Esophagus	90,315 (11.74)	40,285 (7.50)	130,600 (9.99)
	Colorectum	66,249 (8.61)	63,662 (11.85)	129,911 (9.94)
	Female breast	68,253 (8.87)	50,418 (9.38)	118,671 (9.08)
	Liver	66,595 (8.65)	35,419 (6.59)	102,014 (7.81)
	Radiotherapy, chemotherapy	46,515 (6.04)	24,021 (4.47)	70,536 (5.40)
	Uterus	35,992 (4.68)	34,428 (6.41)	70,420 (5.39)
	Lymphoma	23,509 (3.06)	17,234 (3.21)	40,743 (3.12)
	Nasopharynx	20,105 (2.61)	17,172 (3.20)	37,277 (2.85)
	Leukemia	19,719 (2.56)	15,113 (2.81)	34,832 (2.67)
	Bone	13,878 (1.80)	9557 (1.78)	23,435 (1.79)
	Brain	9817 (1.28)	8825 (1.64)	18,642 (1.43)
	Ovary	9444 (1.23)	7441 (1.38)	16,885 (1.29)
	Oral cavity, lip, pharynx	7983 (1.04)	3830 (0.71)	11,813 (0.90)
	Bladder	6432 (0.84)	4352 (0.81)	10,784 (0.83)
	Gallbladder	4887 (0.64)	5038 (0.94)	9925 (0.76)
	Pancreas	5011 (0.65)	4022 (0.75)	9033 (0.69)
	Kidney	5506 (0.72)	3498 (0.65)	9004 (0.69)
	Prostate, testis, penis	4010 (0.52)	4430 (0.82)	8440 (0.65)
	Other (larynx, thyroid, skin, et al)	56,519 (7.35)	48,496 (9.02)	105,015 (8.04)

Table 2 shows the study population composition in the subgroups from 2007 to 2016. Over time, the number of patients gradually increased. The trend in the subgroups was the same as the total.

**Table 2:** Study population composition in the subgroups from 2007 to 2016, n (%)

Year	Coastland			Inland			Total
	Non-surgery	Surgery	Total	Non-surgery	Surgery	Total	
2007	29,372 (4.41)	–	29,372 (3.82)	16,734 (3.58)	–	16,734 (3.11)	46,106 (3.53)
2008	44,136 (6.63)	–	44,136 (5.74)	29,876 (6.40)	–	29,876 (5.56)	74,012 (5.66)
2009	56,783 (8.53)	6 (0.01)	56,789 (7.38)	34,888 (7.47)	2 (0.00)	34,890 (6.49)	91,679 (7.02)
2010	67,439 (10.13)	1126 (1.09)	68,565 (8.91)	44,446 (9.52)	466 (0.66)	44,912 (8.36)	113,477 (8.68)
2011	60,966 (9.16)	14,497 (13.99)	75,463 (9.81)	41,620 (8.91)	9320 (13.21)	50,940 (9.48)	126,403 (9.67)
2012	69,408 (10.42)	14,691 (14.18)	84,099 (10.93)	48,819 (10.46)	9890 (14.02)	58,709 (10.92)	142,808 (10.93)
2013	87,066 (13.08)	17,683 (17.06)	104,749 (13.61)	63,400 (13.58)	11,688 (16.57)	75,088 (13.97)	179,837 (13.76)
2014	93,079 (13.98)	19,224 (18.55)	112,303 (14.59)	63,485 (13.60)	12,395 (17.57)	75,880 (14.12)	188,183 (14.40)
2015	74,593 (11.20)	17,735 (17.11)	92,328 (12.00)	56,924 (12.19)	12,461 (17.67)	69,385 (12.91)	161,713 (12.37)
2016	83,009 (12.47)	18,671 (18.02)	101,680 (13.21)	66,683 (14.28)	14,314 (20.29)	80,997 (15.07)	182,677 (13.98)
Total	665,851 (100.00)	103,633 (100.00)	769,484 (100.00)	466,875 (100.00)	70,536 (100.00)	537,411 (100.00)	1,306,895 (100.00)

### Hospitalization expenses

**Fig. 1:**
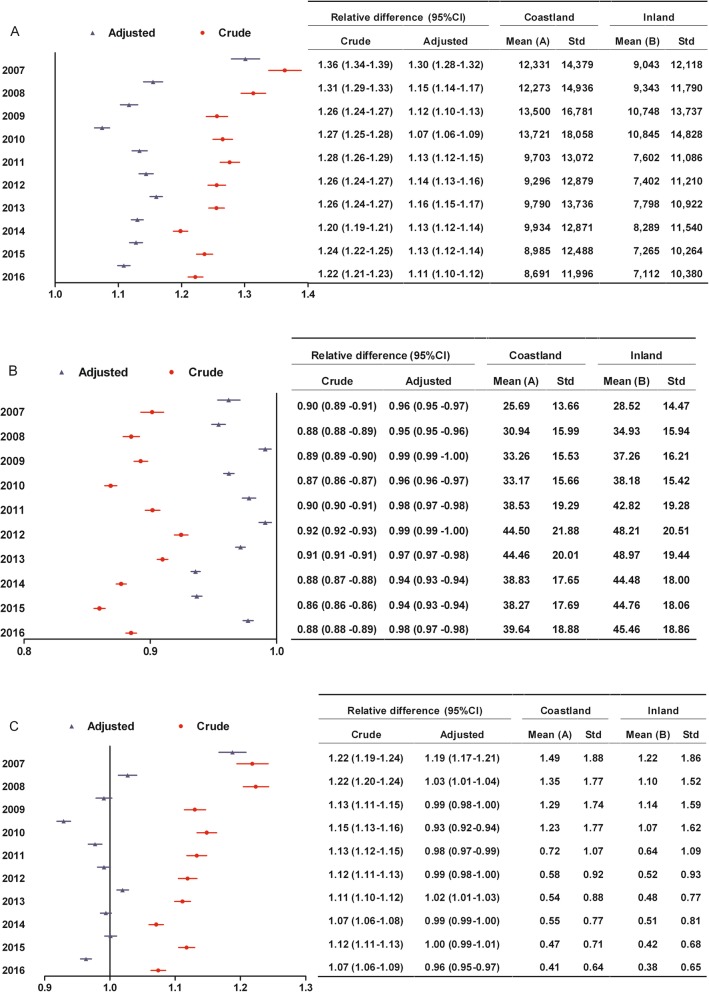
Changes of the disparity between coastland and inland in medical expenditure burden from 2007 to 2016 among patients without surgery. **a** Hospitalization expenses which were all medical expenses incurred during the hospitalization (yuan); **b** Reimbursement ratio (%); **c** Ratio of out-of-pocket expenses to disposable income. Relative difference: Mean (A) divided by Mean (B); CI, Confidence interval; Adjusted: Adjusting for gender, age, tumor site and hospital level; Std, Standard deviation

Among patients without surgery, coastland patients had higher hospitalization expenses than inland patients during the study period. The hospitalization expenses between 2007 and 2010 as a whole were higher than those between 2011 and 2016. The relative difference (95% CI) between coastland and inland in hospitalization expenses was moving closer to 1.0 from 2007 to 2010, implying that the disparity between two areas significantly narrowed. The hospitalization expenses slightly increased from 2011 to 2014 and obviously decreasd from 2014 to 2016. The range of change was similar between coastland and inland from 2011 to 2016, implying that the disparity between two areas in hospitalization expenses did not significantly change (Fig. 1a).

**Fig. 2:**
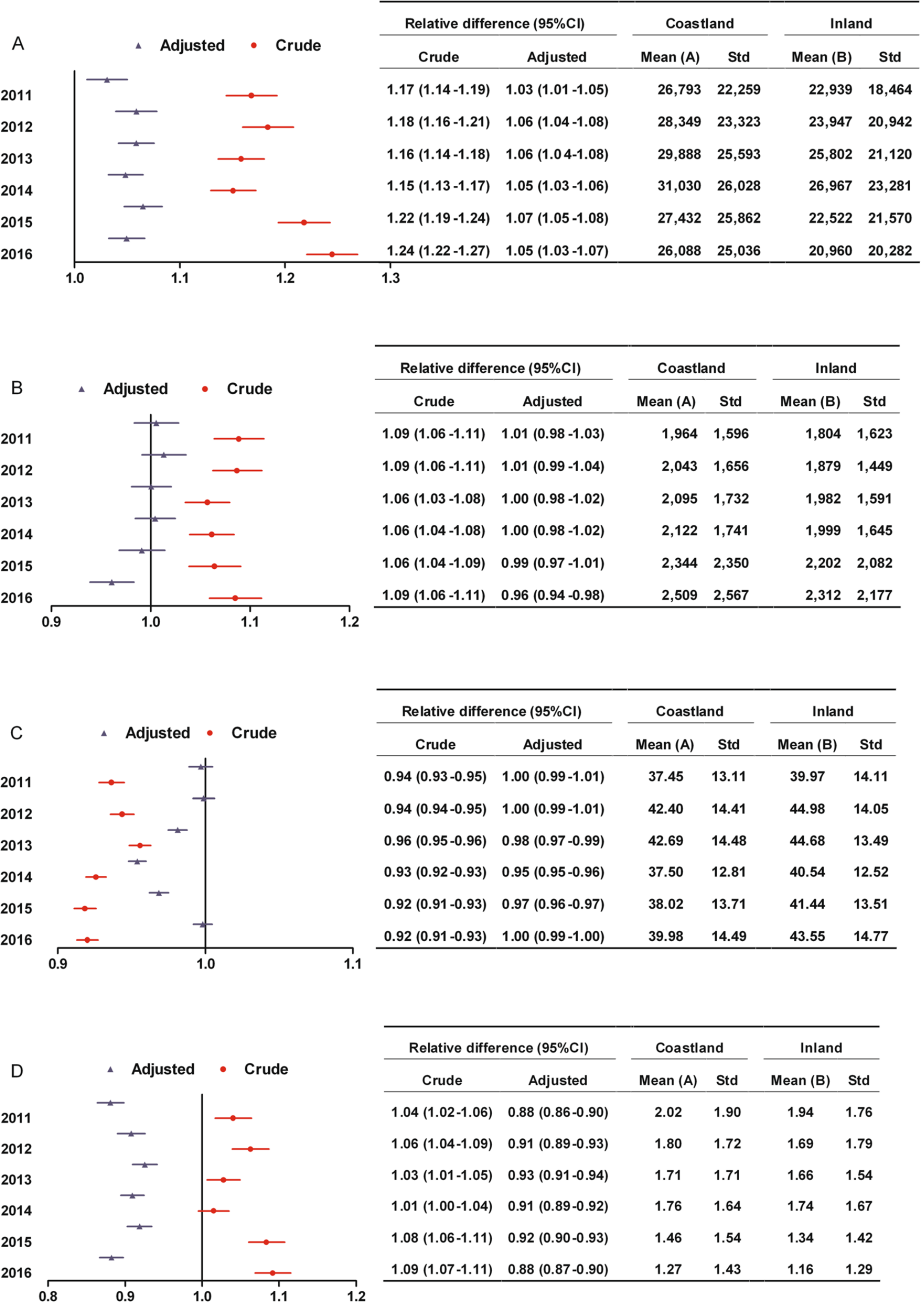
Changes of the disparity between coastland and inland in medical expenditure burden from 2011 to 2016 among patients with surgery. **a** Hospitalization expenses which were all medical expenses incurred during the hospitalization (yuan); **b** Surgery expenses (yuan); **c** Reimbursement ratio (%); **d** Ratio of out-of-pocket expenses to disposable income. Relative difference: Mean (A) divided by Mean (B); CI, Confidence interval; Adjusted: Adjusting for gender, age, tumor site and hospital level; Std, Standard deviation

Among patients with surgery, coastland patients had higher hospitalization expenses than inland patients. The hospitalization expenses were highest in 2014 and lowest in 2016. The range of change was similar between coastland and inland from 2011 to 2016, implying that the disparity between two areas in hospitalization expenses did not significantly change (Fig. 2a).

### Surgery expenses

Figure 2b shows the change of the disparity between coastland and inland in surgery expenses from 2011 to 2016. The differences between coastland and inland patients in surgery expenses were all not statistically significant between 2011 and 2015. The surgery expenses were lowest in 2011 and highest in 2016. The range of increase for surgery expenses was similar between coastland and inland from 2011 to 2015, implying that the disparity between two areas did not significantly change. The surgery expenses of inland patients became higher than those of coastland patients in 2016

### Reimbursement ratio

Among patients without surgery, coastland patients had lower reimbursement ratio than inland patients and the highest reimbursement ratio was still below 50% during the study period. The range of increase for reimbursement ratio was similar between coastland and inland from 2007 to 2010, implying that the disparity between two areas did not significantly change. The relative difference (95% CI) between coastland and inland in reimbursement ratio was moving away from 1.0 from 2011 to 2014 and moving close to 1.0 from 2014 to 2016, as a result that the disparity between two areas in reimbursement ratio went back to the level in 2011 (Fig. 1b).

Among patients with surgery, coastland patients had lower reimbursement ratio than inland patients between 2013 and 2015. The highest reimbursement ratio was even below 45% during the study period. The relative difference (95% CI) between coastland and inland in reimbursement ratio was moving away from 1.0 from 2011 to 2014 and moving close to 1.0 from 2014 to 2016, as a result that the disparity between two areas in reimbursement ratio went back to the level in 2011 (Fig. 2c).

### Ratio of out-of-pocket expenses to disposable income

Among patients without surgery, the OOP ratio decreased from 2007 to 2010 but was still higher than 1.0 in 2010. The relative difference (95% CI) between coastland and inland in OOP ratio was moving closer to 1.0 from 2007 to 2009, implying that the disparity between two areas significantly narrowed. The OOP ratio of coastland patients became lower than or no statistical difference from that of inland patients started from 2010. The OOP ratio continuously decreased from 0.7 in 2011 to 0.4 in 2016. The range of decrease for OOP ratio was similar between coastland and inland from 2011 to 2016, implying that the disparity between two areas did not significantly change (Fig. 1c).

Among patients with surgery, coastland patients had lower OOP ratio than inland patients during the study period. The OOP ratio was lowest, but the out-of-pocket expenses still exceeded annual disposable income in 2016. The range of decrease for OOP ratio was similar between coastland and inland from 2011 to 2016, implying that the disparity between two areas did not significantly change (Fig. 2d).

### Stratified analysis by household income situation

The above analyses were repeated for non-low income and low-income patients separately. Low-income patients had lower medical expenses and OOP ratio, and higher reimbursement ratio than non-low income patients. The disparity between coastland and inland in medical expenditure burden depended on the household income situation. For non-low income patients, the trend of the disparity between coastland and inland in medical expenditure burden was the same as the total (Supplementary Figure 1 and Supplementary Figure 2).

**Fig. 3:**
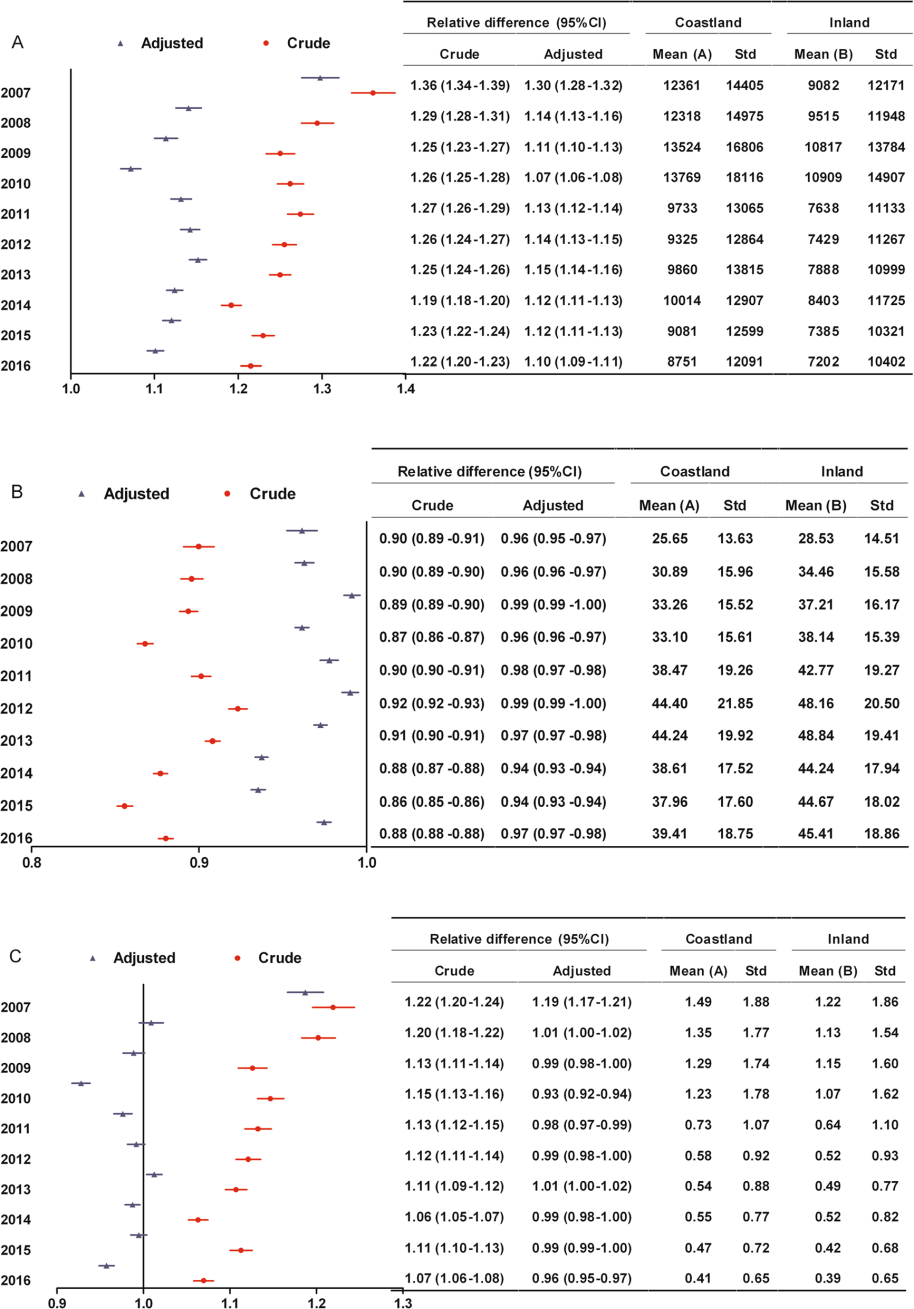
Changes of the disparity between coastland and inland in medical expenditure burden from 2007 to 2016 among low-income patients without surgery. **a** Hospitalization expenses which were all medical expenses incurred during the hospitalization (yuan); **b** Reimbursement ratio (%); **c** Ratio of out-of-pocket expenses to disposable income. Relative difference: Mean (A) divided by Mean (B); CI, Confidence interval; Adjusted: Adjusting for gender, age, tumor site and hospital level; Std, Standard deviation

**Fig. 4:**
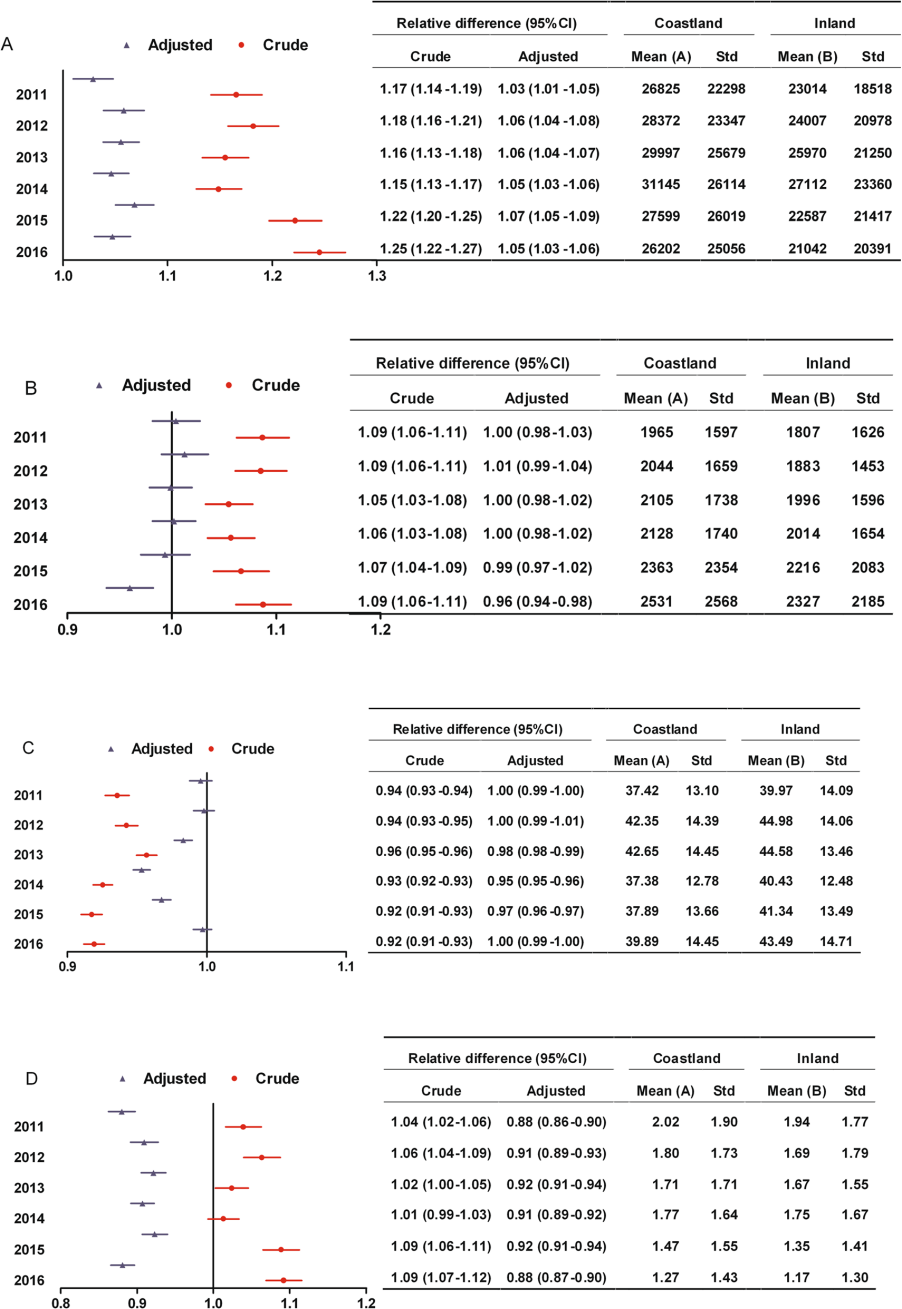
Changes of the disparity between coastland and inland in medical expenditure burden from 2011 to 2016 among low-income patients with surgery. **a** Hospitalization expenses which were all medical expenses incurred during the hospitalization (yuan); **b** Surgery expenses (yuan); **c** Reimbursement ratio (%); D: Ratio of out-of-pocket expenses to disposable income. Relative difference: Mean (A) divided by Mean (B); CI, Confidence interval; Adjusted: Adjusting for gender, age, tumor site and hospital level; Std, Standard deviation

For low-income patients, the results were different from the total, as following:

1. **Hospitalization expenses:** The differences between coastland and inland patients with surgery in hospitalization expenses were not statistically significant. Whether among patients without or with surgery, the range of change was similar between coastland and inland over time, implying that the disparity between two areas in hospitalization expenses did not significantly change (Figs. 3a and 4a);
2. **Surgery expenses:** There were no statistical differences in surgery expenses between coastland and inland patients during the study period. The range of change was similar between coastland and inland from 2011 to 2016, implying that the disparity between two areas in surgery expenses did not significantly change (Fig. 4b);
3. **Reimbursement ratio:** Among patients without surgery, the reimbursement ratio increased among coastland patients and decreased among inland patients, and the relative difference (95% CI) between coastland and inland in reimbursement ratio was moving closer to 1.0 from 2008 to 2010, implying that the disparity between two areas significantly narrowed. The range of change was similar between coastland and inland from 2011 to 2016, implying that the disparity between two areas in reimbursement ratio did not significantly change (Fig. 3b).

Among patients with surgery, the reimbursement ratio of coastland patients was higher than that of inland patients before 2013 and became no statistical difference from that of inland patients started in 2014. The relative difference (95% CI) between coastland and inland in reimbursement ratio was moving closer to 1.0 from 2011 to 2016, implying that the disparity between two areas significantly narrowed (Fig. 4c).

(4) **Ratio of out-of-pocket expenses to disposable income:** Whether among patients without or with surgery, the differences between coastland and inland patients in OOP ratio were not statistically significant in most years. The range of change was similar between coastland and inland over time, implying that the disparity between two areas in OOP ratio did not significantly change (Figs. 3c and 4d).

## Discussion

The data of medical expenses from the medical records of inpatients with NRCMS in Fujian Province showed that the NRCMS had made some achievements but its protective effect was limited. The hospitalization expenses and OOP ratio had decreased, and reimbursement ratio had increased for rural patients with malignant tumor from 2007 to 2016. However, the reimbursement ratio was still less than 50% and the out-of-pocket expenses still exceeded annual disposable income among patients with surgery in 2016. The disparity between coastland and inland in medical expenditure burden had statistically narrowed from 2007 to 2010, but the disparity between two areas did not significantly change from 2011 to 2016 whether among patients without or with surgery.

The Statistical Yearbook of Fujian province in 2017 reported that the number of rural population had gradually declined from 17.56 million in 2007 to 14.10 million in 2016 due to rural urbanization [18]. Conversely, this study displayed that the number of rural inpatients with malignant tumor gradually increased during the same period. To some extent, the inconformity indirectly reflected that the prevalence rate of malignant tumor continuously increased. With the development of economy, the per capita disposable income and health awareness of residents increased so that the residents would go to hospital when they were ill [19, 20]. In addition, the unceasing improvement of NRCMS promoted the participation rate increasing which also resulted in the rise of hospital admissions [21, 22].

The State Council of China increased investments to deepen the health-care system reform between 2009 and 2011 [23, 24]. This reform focused on five aspects: basic medical security system, essential medicine system, grassroots health care services system, equity of public health service and public hospitals reform, which aimed to reduce the disease economic burden and improve the accessibility and equity of health service for residents [25]. Although the hospitalization expenses increased, the reimbursement ratio increased and OOP ratio decreased, and the disparity between coastland and inland in medical expense indicators had statistically narrowed from 2007 to 2010. This study also showed that hospitalization expenses and OOP ratio rapidly declined starting from 2011 and reimbursement ratio was highest in 2012. These results verified the achievements which had been made by the deepening reform on health-care.

The hospitalization expenses and OOP ratio had slight rise and reimbursement ratio had about 5% reduction in 2014 compared to the previous year. It may be related to the positive effect of deepening reform on health-care gradually diminishing over time [26]. With some problems which occurred during the deepening reform stage solved and the reform further deepened [27], the hospitalization expenses and OOP ratio decreased and the reimbursement ratio increased again from 2015. However, the disparity between coastland and inland in medical expense indicators did not significantly change from 2011 to 2016.

High-level hospitals with high-quality medical resources, including sophisticated experts, advanced equipment, high-quality medical service and so on, were mainly concentrated in coastland, accompanied with higher medical expenses than those in inland [27, 28]. Some inland patients who needed surgery and had better economic condition would tend to be admitted in high-level hospitals, expecting higher likelihood of survival. In this case, they faced higher consumption level which may be the reason the surgery expenses obviously increased. In contrast to increased surgery expenses, the hospitalization expenses decreased which may be associated with the positive effect of National Essential Medicines Policy on reducing medicines expenses [29, 30].

The inland patients had higher reimbursement ratio than coastland patients that may be due to the following two reasons: First, inland patients would tend to choose the treatments and pharmaceuticals which could be covered by medical insurance reimbursement directory. Second, the government took a series of measures for low-level hospitals to reallocate the medical resources, in order to encourage the patients to be admitted in hospitals which located in their place of household [31]. One of the measures is to decrease deductible and increase reimbursement ratio in low-level hospital. The average level of deductible (reimbursement ratio) was 200 yuan (70–90%) in township hospitals, 500 yuan (60–80%) in county hospitals, 800 yuan (50–70%) in municipal hospitals and 1500 yuan (45–60%) in provincial hospitals.

Although NRCMS played an important role in reducing medical expenses and increasing reimbursement ratio for rural inpatients with malignant tumor, their medical expenditure burden was still heavy. This study showed that out-of-pocket expenses were in decline from 2007 to 2016, but they still accounted for more than 55% of hospitalization expenses in 2016. It was obviously different from that the proportion of individual medical expenditure decreased from 44.05% in 2007 to 28.78% in 2016 which was reported by China Health Statistical Yearbook in 2017 [32]. Nevertheless, the difference could be explained. Most of rural resident had no regular physical examination for economic reasons. They were usually diagnosed as malignant tumor in middle and / or late stage with serious condition [33, 34]. In addition, the treatment of malignant tumor was more complicated and the treatment cycle was longer than the other diseases, and fewer anticarcinogens were covered by medical insurance reimbursement directory [35]. Therefore, the proportion of individual medical expenses for patients with malignant tumor was much higher than the average level. Furthermore, per capita annual disposable income of Fujian rural residents increased year by year, but the OOP ratio was about 40% among patients without surgery and even 1.2 times among patients with surgery in 2016. The inland patients had higher OOP ratio than coastland patients. These findings implied that it was absolutely a catastrophic event if only one person in a family suffered from malignant tumor in China, especially for inland families.

Low-income residents whose household per capita income were below the local minimum living standard were admitted to participate in the basic medical insurance system with lower insurance premium than non-low income residents [23, 25]. When they get illness, they can be safeguarded by medical assistance system except for basic medical insurance system with lower deductible and higher reimbursement ceiling than non-low income patients [25, 31, 36]. Besides, low-income patients would tend to choose conservative treatment and the treatments which could be covered by medical insurance reimbursement directory. These were consistent with the results of this study that low-income patients had lower medical expenses and OOP ratio, and higher reimbursement ratio. The difference between coastland and inland low-income patients in OOP ratio was not statistically significant, implying that the medical expenditure burden was similar between two areas.

Several issues should be considered in this study. First, the trend of disparity between coastland and inland in medical expenditure burden for patients with surgery from 2011 to 2016 was assessed due to few data before 2011. Second, because of the long time span and purpose to determine whether the changes of disparity differed across surgery or not, the trend of disparity between coastland and inland were reported in two phases: from 2007 to 2010 and from 2011 to 2016. Third, the findings of multivariate analyses which were adjusted for gender, age, tumor site and hospital level were only described in the Results section; Finally, two limitations should be considered: the individual income could not be assessed limited by the data source. However, the per capita annual disposable income of each county was using which could also reflect the income situation of patients to some degree; Because medical record management systems differed across different hospitals, a patient whom may be admitted to different hospitals across regions could not be traced.

## Conclusions

Under the effect of NRCMS, the hospitalization expenses and OOP ratio decreased, and reimbursement ratio increased for rural inpatients with malignant tumor from 2007 to 2016. The disparity between coastland and inland in medical expenditure burden had statistically narrowed from 2007 to 2010, but the disparity between two areas did not significantly change from 2011 to 2016 whether among patients without or with surgery. As a whole, the inland patients had heavier medical expenditure burden than coastland patients. Because of economic factors and medical assistance policies, the medical expenditure burden was similar between coastland and inland low-income patients.

## Supplementary information

**Additional file 1: Supplementary Table 1.** The consumer price index and real price from 2007 to 2016.

**Additional file 2 **: **Supplementary Figure 1.** Changes of the disparity between coastland and inland in medical expenditure burden from 2007 to 2016 among non-low income patients without surgery. A: Hospitalization expenses which were all medical expenses incurred during the hospitalization (yuan); B: Reimbursement ratio (%); C: Ratio of out-of-pocket expenses to disposable income. Relative difference: Mean (A) divided by Mean (B); CI, Confidence interval; Adjusted: Adjusting for gender, age, tumor site and hospital level; Std, Standard deviation.

**Additional file 3 **: **Supplementary Figure 2.** Changes of the disparity between coastland and inland in medical expenditure burden from 2011 to 2016 among non-low income patients with surgery. A: Hospitalization expenses which were all medical expenses incurred during the hospitalization (yuan); B: Surgery expenses (yuan); C: Reimbursement ratio (%); D: Ratio of out-of-pocket expenses to disposable income. Relative difference: Mean (A) divided by Mean (B); CI, Confidence interval; Adjusted: Adjusting for gender, age, tumor site and hospital level; Std, Standard deviation.

## Data Availability

The datasets used in this study are available from the corresponding author on reasonable request.

## Abbreviations

NRCMS: New Rural Cooperative Medical Scheme
ICD-10: International Classification of Diseases, Tenth Revision
OOP ratio: Ratio of out-of-pocket expenses to disposable income
CPI: Consumer price index
CI: Confidence intervals
