# Supplementary material for: Trend of disparity between coastland and inland in medical expenditure burden for rural inpatients with malignant tumor in southeast of China from 2007 to 2016
Source: BMC Cancer. 2020 Apr 7;20:295. doi: 10.1186/s12885-020-06769-6 (PMC7140354; doi:10.1186/s12885-020-06769-6)
Supplement: Supplementary file 1 — Additional file 1: Supplementary Table 1. The consumer price index and real price from 2007 to 2016. [file 12885_2020_6769_MOESM1_ESM.doc]

Supplementary Table 1. The consumer price index and real price from 2007 to 2016.

| year | CPI | Real price (yuan) |
| --- | --- | --- |
| 2007 | 493.6 | 100.0 |
| 2008 | 522.7 | 94.4 |
| 2009 | 519.0 | 95.1 |
| 2010 | 536.1 | 92.1 |
| 2011 | 565.0 | 87.4 |
| 2012 | 579.7 | 85.2 |
| 2013 | 594.8 | 83.0 |
| 2014 | 606.7 | 81.4 |
| 2015 | 615.2 | 80.2 |
| 2016 | 627.5 | 78.7 |

CPI, Consumer price index, reference as CPI=100 in 1978; The real price in 2008 was calculated as: 100 yuan × (493.6/522.7) = 94.4 yuan, and so on.

Supplementary Figure 1. Changes of the disparity between coastland and inland in medical expenditure burden from 2007 to 2016 among non-low income patients without surgery. A: Hospitalization expenses which were all medical expenses incurred during the hospitalization (yuan); B: Reimbursement ratio (%); C: Ratio of out-of-pocket expenses to disposable income. Relative difference: Mean (A) divided by Mean (B); CI, Confidence interval; Adjusted: Adjusting for gender, age, tumor site and hospital level; Std, Standard deviation.

Supplementary Figure 2. Changes of the disparity between coastland and inland in medical expenditure burden from 2011 to 2016 among non-low income patients with surgery. A: Hospitalization expenses which were all medical expenses incurred during the hospitalization (yuan); B: Surgery expenses (yuan); C: Reimbursement ratio (%); D: Ratio of out-of-pocket expenses to disposable income. Relative difference: Mean (A) divided by Mean (B); CI, Confidence interval; Adjusted: Adjusting for gender, age, tumor site and hospital level; Std, Standard deviation.
